# Effect of later cord clamping on umbilical cord blood gas in term neonates of diabetic mothers: a randomized clinical trial

**DOI:** 10.1186/s12887-022-03170-z

**Published:** 2022-03-01

**Authors:** Hailing Shao, Yehui Lan, Yiyu Qian, Ruyang Chen, Lingli Peng, Ying Hua, Xiaomei Wang

**Affiliations:** 1 Department of Obstetrics and Gynecology, the Second Affiliated Hospital of Wenzhou Medical University, 325027 Wenzhou, China; 2 Department of Obstetrics and Gynecology, Wenzhou Central Hospital, 325000 Wenzhou, China; 3 Department of Gynecology, Wenzhou People Hospital, 325000 Wenzhou, China

**Keywords:** Later cord clamping, Diabetic pregnancy, Umbilical cord blood gas

## Abstract

**Objective:**

To evaluate the effect of later cord clamping (LCC) on umbilical arterial blood gas in neonates of diabetic mothers.

**Methods:**

This prospective study included a group of 160 diabetic mothers (DM) whose neonates were randomized to immediate cord clamping (ICC) (≤ 15 s after birth) or LCC (≥ 30 s after birth), and a group of 208 non-diabetic mothers (NDM) whose neonates were randomized to ICC or LCC as a reference. Cord arterial pH, base excess (BE), bicarbonate (HCO_3_^−^), partial pressure of carbon dioxide (pCO_2_), partial pressure of oxygen (pO_2_), lactate, hemoglobin, hematocrit and glucose were compared among groups.

**Results:**

In neonates of DM, there was no significant difference in cord arterial pH between the ICC and LCC group. LCC of ≥ 30 s decreased umbilical arterial HCO_3_^−^ and BE and increased lactate (ICC versus LCC, HCO_3_^−^: 24.3 (22.7, 25.8) versus 23.7 (22.3, 24.7) mmol/L, *P* = 0.01; BE: -2.70 (-4.80, -1.50) versus − 3.72 (-5.66, -2.36) mmol/L, *P* = 0.006; lactate: 2.1 (1.6, 3.7) versus 2.7 (2.1, 4.3) mmol/L, *P* = 0.005), without the alterations of pCO_2_, pO_2_, hemoglobin, hematocrit and glucose. Similar results were found in neonates of NDM (ICC versus LCC, HCO_3_^−^: 24.3 (23.1, 25.7) versus 23.5 (22.3, 24.8) mmol/L, *P* = 0.01; BE: -2.39 (-3.73, -1.51) versus − 3.40 (-4.73, -1.91) mmol/L, *P* = 0.001; lactate: 2.2 (1.9, 3.3) versus 2.5 (2.0, 4.3) mmol/L, *P* = 0.01), except for the higher level of hemoglobin in the LCC group. The majority of diabetic mothers (ICC: 92.0%; LCC: 91.8%) had good blood glucose control. No differences were observed in acid-base status and glucose between neonates of DM and neonates of NDM in both ICC and LCC, but hemoglobin and hematocrit were elevated after ICC in neonates of DM compared to neonates of NDM.

**Conclusions:**

Later cord clamping of ≥ 30 s resulted in a tendency towards metabolic acidosis of umbilical arterial blood in neonates of DM and NDM. Umbilical arterial blood gas parameters at birth were similar in neonates of DM and NDM.

**Trial registration:**

ClinicalTrials.gov: NCT04369313; date of registration: 30/04/2020 (retrospectively registered).

## Background

Umbilical cord blood gas analysis, a valuable and reliable method, is used for assessing the acid-base status of neonates [1–3]. Umbilical artery carries blood from fetus to placenta, thus umbilical arterial blood gas is more reflective of the fetus status, which is widely recommended in high-risk deliveries [1]. Umbilical arterial blood gas analysis is an essential criterion for neonatal hypoxic ischemic encephalopathy [4]. Neonates with 5-minute Apgar scores of ≥ 7, umbilical artery pH ≤ 7.00 combined with BE ≥ 12 mmol/L were more likely to develop respiratory distress syndrome (RDS) [5].

The American College of Obstetricians and Gynecologists (ACOG) recommended a delay of cord clamping for at least 30–60 s (s) in neonates, because it improved hemoglobin levels and iron stores [6]. To date, several studies had investigated the influence of later cord clamping (LCC) on umbilical cord blood gas in low-risk pregnancy. LCC led to a tendency of acidemia in umbilical cord blood, presenting as decreased pH, base excess (BE) and bicarbonate (HCO_3_^−^) and increased lactate and partial pressure of carbon dioxide (pCO_2_) [7–9]. In contrast, several studies demonstrated that arterial and venous cord blood pH was unaffected after LCC [10–12].

Pregnancy complicated with diabetes mellitus is associated with short-term adverse implications of neonates, including respiratory distress syndrome, hypoglycemia, polycythemia, hyperbilirubinemia, and even perinatal death [13]. Due to fetal hyperinsulinemia and subsequent lung surfactant synthesis, neonates of diabetic mothers (DM) are more susceptible to intrapartum hypoxia [13]. A retrospective study showed that, under the condition of non-reassuring fetal heart rate, umbilical arterial blood pH decreased by 0.035 (7.250–7.215) in neonates of DM than that in neonates of non-diabetic mothers (NDM) [14]. Besides, compared with healthy newborns, neonates of DM were at an approximately five-fold risk of admission to intensive care unit and a 2–9 fold risk of 5-min Apgar score of < 7 [15, 16].

Currently, no studies have described the effect of LCC on umbilical cord blood gas in neonates of diabetic mothers. The prevalence of gestational diabetes mellitus has been on the rise in recent years. Therefore, in this study, we identified the changes of umbilical arterial blood gas and hematological parameters following LCC of ≥ 30 s in neonates of DM and NDM without the need for immediate resuscitation.

## Methods

This was a prospective study conducted in the Second Affiliated Hospital of Wenzhou Medical University from September 2019 to September 2020 (clinicaltrials.gov: NCT04369313, ClinicalTrials.gov: NCT04369313; date of registration: 30/04/2020). This study was supported by the Research Ethics Committee of our hospital (approval number: L-2019-13). Pregnant women all signed written informed consent before delivery. Delayed cord clamping was introduced in this hospital in 2017. In this study, the clamping time in the immediate cord clamping (ICC) group was less than 15 s after birth, and it was more than 30 s in the LCC group.

All diabetic mothers with a singleton pregnancy at term were screened for eligibility when they were admitted into our hospital for vaginal delivery or selective cesarean section. Pregnancy complicated with diabetes included gestational diabetes mellitus (GDM) and pregestational diabetes mellitus (PGDM). GDM was screened by 75 g oral glucose tolerance test (OGTT) at 24–28 weeks of gestation. Fasting plasma glucose (FPG) ≥ 5.1 mmol/L, or 1 h (h) plasma glucose ≥ 10.0mmol/L, or 2 h plasma glucose ≥ 8.5 mmol/L was diagnostic criteria for GDM. Women at high risk for diabetes were tested for FPG and HbA1C at the first prenatal visit. FPG ≥ 7.0 mmol/L or HbA1C ≥ 6.5% was considered as PGDM. Well-controlled blood glucose was defined as an HbA1C level < 6.0% in late pregnancy. Mothers with other pregnancy complications (hypertension disorders, intrahepatic cholestasis of pregnancy, maternal fever, multiple pregnancy, preterm labor, post-term pregnancy, emergency cesarean section, abnormal fetal presentation) and the neonates with birth weight < 2500 g, Apgar score of ≤ 7, neonatal malformation, suspicious fetal distress, neonatal resuscitation were all excluded. In addition, mothers were withdrawn from the study if cord blood collection failed and blood gas parameters were missed. Non-diabetic mothers at term were also recruited consecutively.

Diabetic women were randomly assigned to ICC or LCC at a ratio of 1:1, by using a random number sequence generator. Opaque envelopes with an A card or a B card inside were pre-prepared and then arranged according to the random sequence. The women who drew the A card were assigned to the ICC group, and the women who drew the B card were assigned to the LCC group. Non-diabetic mothers were also randomized according to the method above.

Because the procedure of umbilical cord clamping was carried out by researchers and mothers were always clearly conscious, the group allocation was not possible to be masked to the mothers, the researchers performing the intervention and the assistant recording the clamping time. However, the staff responsible for the blood gas analysis and data collection was blind to patient’s allocation.

Before the cord was clamped, neonates with vaginal delivery were placed on the abdomen of mothers, and neonates with cesarean section were placed between the legs of mothers. The clamping time of each group was recorded by an assistant. The trial should be discontinued immediately if any rescue intervention was required for neonates or mothers.

Three vascular forceps were used to clamp the umbilical cord. The first forcep was placed at the unbilical cord 5 cm from the fetal side, the second forcep was at 5–10 cm from the first forcep, and the third forcep was at the umbilical cord 10 cm from the placenta. The umbilical cord was cut between the first and the second vascular forceps. At least 0.5 ml of umbilical arterial blood was extracted between the second and the third vascular forceps by a pre-heparinized syringe. Blood gas analysis and the test of hemoglobin (Hb), hematocrit (Hct) and glucose in umbilical arterial blood were performed using the automated blood gas analyser (Cobas b 123, Roche) within 15 min.

According to the previous study, umbilical arterial blood pH was 7.28 ± 0.07 [12]. A sample size of 66 in each group achieved 90% power to detect a 0.04 decrease of arterial pH in the DCC group with a two-sided significance level of 0.05. Considering that unqualified cases needed to be excluded from the trial, we increased the number of cases in NP groups to 130 and DP groups to 90. The primary outcome was the change of umbilical cord arterial pH. The secondary outcomes were HCO_3_^−^, BE, lactate, partial pressure of oxygen (pO_2_) and pCO_2_ of umbilical arterial blood, neonatal hemoglobin (Hb), hematocrit (Hct) and blood glucose.

The data were analyzed by SPSS 25.0 software. The comparisons of continuous variables with normal distributions (mean ± standard deviation) were conducted by Student t-test or one-way ANOVA. Mann-Whitney U test was used to compare continuous variables without normal distribution (median with interquartile range), and Pearson’s Chi-square test was used to compare categorical variables. A two-sided *P*-value < 0.05 meaned statistically significant.

## Results

**Fig. 1:**
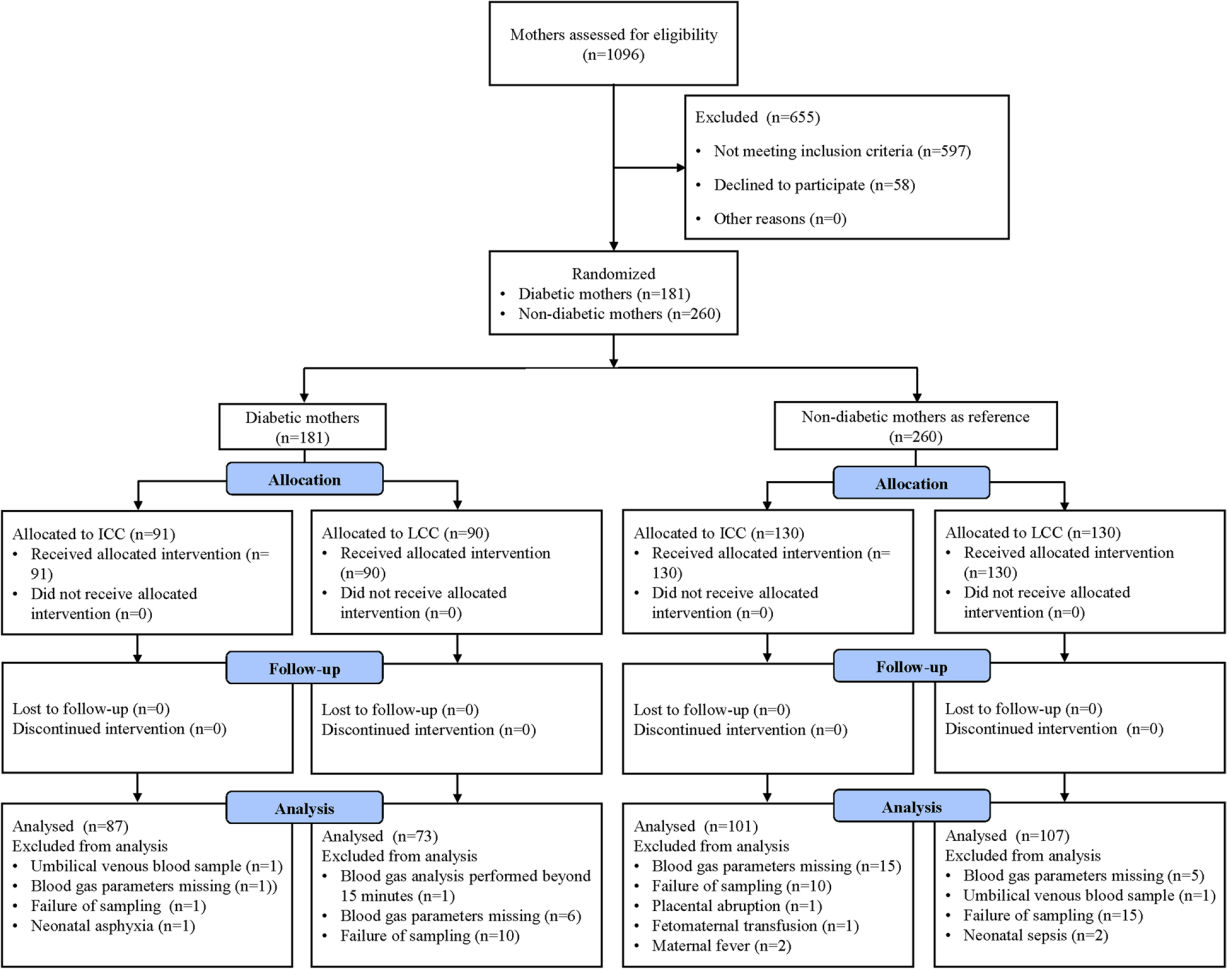
Study flow chart

One thousand ninety-six pregnant women were assessed for eligibility from September 2019 to September 2020, 597 of whom were excluded for not meeting the inclusion criteria and 58 of whom declined to participate in this study. Then, 441 pregnant women were enrolled, of whom 73 pregnancies were excluded from the analysis. Finally, among diabetic mothers, 87 cases received ICC and 73 cases received LCC, and among non-diabetic mothers, 101 cases received ICC and 107 cases received LCC (Fig. 1).

As shown in Table 1, no differences were found in the demographic characteristics of mothers and neonates among the four groups. The majority of diabetic mothers had good blood glucose control (ICC: 92.0%; LCC: 91.8%). The median clamping time was 6 s in DM (ICC) and 44 s in DM (LCC), 6 s in NDM (ICC), and 45 s in NDM (LCC), respectively (Table 2).

**Table 1:** Demographic characteristics of mothers and neonates

	DM			NDM		*P* ^*^
	ICC *N *= 87	LCC *N* = 73		ICC *N *= 101	LCC *N* = 107	
**Mothers**						
Age (years)	31.0 ± 3.9	30.5 ± 4.9		29.7 ± 5.2	30.2 ± 4.4	0.30
BMI (Kg/m^2^)	27.2 ± 3.4	27.1 ± 3.0		27.1 ± 3.6	26.4 ± 2.7	0.31
Prenatal Hb (g/L)	119.5 ± 11.2	121.0 ± 12.0		117.9 ± 11.4	119.3 ± 11.7	0.38
Blood glucose control, n (%)						
Well-controlled	80 (92.0)	67 (91.8)		-	-	0.97 ^a^
Poorly-controlled	7 (8.0)	6 (8.2)		-	-	
**Neonates**						
Gestational age (weeks)	38.8 ± 0.8	39.0 ± 1.0		39.0 ± 0.8	39.0 ± 0.9	0.52
Delivery method, n (%)						
Vaginal delivery	32 (36.8)	31 (42.5)		32 (31.7)	33 (30.8)	0.36
Cesarean section	55 (63.2)	42 (57.5)		69 (68.3)	74 (69.2)	
Neonatal sex, n (%)						
Male	49 (56.3)	37 (50.7)		44 (43.6)	60 (56.1)	0.24
Female	38 (43.7)	36 (49.3)		57 (56.4)	47 (43.9)	
Birthweight (g)	3321.9 ± 394.9	3394.1 ± 413.0		3290.5 ± 361.2	3378.4 ± 374.0	0.23
Body length (cm)	50 (49, 50)	50 (50, 50)		50 (49, 50)	50 (49, 50)	0.26
1-min Apgar score	10 (10, 10)	10 (10, 10)		10 (10, 10)	10 (10, 10)	0.32
5-min Apgar score	10 (10, 10)	10 (10, 10)		10 (10, 10)	10 (10, 10)	0.79

*NDM* non-diabetic mothers, *DM* diabetic mothers, *ICC* immediate cord clamping, *LCC* later cord clamping

*BMI* body mass index, *Hb* hemoglobin

Data were given as mean ± SD, n (%) or median (IQR)

^*^ Chi-square test for categorical variables, Mann–Whitney U-test or student t-test for quantitative variables; the comparisons were conducted among four groups

^a^ The comparisons were conducted between ICC and LCC from DM

**Table 2:** Comparisons of umbilical arterial blood gas analysis between groups

	DM		NDM		DM	NDM	DM vs NDM	
	ICC	LCC	ICC	LCC	*P* ^*^			
	*N* = 87	*N* = 73	*N* = 101	*N* = 107	ICC vs LCC	ICC vs LCC	ICC	LCC
Cord clamping time (seconds)	6 (4, 9)	44 (37, 55)	6 (4, 9)	45 (38, 56)	< 0.001	< 0.001	0.82	0.83
pH	7.309 (7.255, 7.330)	7.303 (7.245, 7.328)	7.303 (7.280, 7.327)	7.305 (7.273, 7.322)	0.41	0.37	0.74	0.89
pCO_2_ (mmHg)	50.1 (45.8, 54.1)	49.2 (45.7, 55.3)	49.5 (47.4, 53.8)	49.5 (45.2, 54.3)	0.32	0.42	0.89	0.84
pO_2_ (mmHg)	19.8 (17.0, 23.2)	21.1 (17.1, 24.1)	19.1 (16.0, 24.1)	20.4 (16.5, 24.5)	0.25	0.30	0.92	0.85
HCO_3_^-^ (mmol/L)	24.3 (22.7, 25.8)	23.7 (22.3, 24.7)	24.3 (23.1, 25.7)	23.5 (22.3, 24.8)	0.01	0.01	0.87	0.74
BE (mmol/L)	-2.70 (-4.80, -1.50)	-3.72 (-5.66, -2.36)	-2.39 (-3.73, -1.51)	-3.40 (-4.73, -1.91)	0.006	0.001	0.22	0.08
Lactate (mmol/l)	2.1 (1.6, 3.7)	2.7 (2.1, 4.3)	2.2 (1.9, 3.3)	2.5 (2.0, 4.3)	0.005	0.01	0.61	0.55
Hb (g/dl)	15.2 (14.0, 16.1)	15.4 (14.4, 16.4)	14.6 (13.9, 15.4)	14.9 (13.9, 16.3)	0.43	0.02	0.007	0.33
Hct (%)	44.0 (39.8, 47.6)	44.8 (41.0, 48.6)	42.5 (39.9, 44.8)	43.1 (40.1, 47.3)	0.20	0.06	0.02	0.09
Glucose (mmol/L)	3.8 (3.4, 4.7)	3.8 (3.3, 4.8)	3.7 (3.4, 4.6)	3.6 (3.2, 4.4)	0.85	0.35	0.62	0.16

*NDM *non-diabetic mothers, *DM *diabetic mothers, *ICC *immediate cord clamping, *LCC *later cord clamping

^*^ Mann–Whitney U-test for quantitative variables

### LCC versus ICC in neonates of diabetic mothers

In comparison with ICC, the umbilical arterial blood HCO_3_^−^ and BE decreased markedly in LCC, while the lactate level increased (LCC versus ICC, HCO_3_^−^: 24.3 (22.7, 25.8) versus 23.7 (22.3, 24.7) mmol/L; BE: -2.70 (-4.80, -1.50) versus − 3.72 (-5.66, -2.36) mmol/L; lactate: 2.1 (1.6, 3.7) versus 2.7 (2.1, 4.3) mmol/L). Other umbilical arterial blood gas parameters (pH, pCO_2_, pO_2_, Hb, Hct and glucose) did not differ significantly between LCC and ICC (Table 2).

### LCC versus ICC in neonates of non-diabetic mothers

Comparisons of umbilical arterial blood gas parameters in NDM showed that HCO_3_^−^ and BE were significantly decreased, but lactate and Hb were markedly increased in the LCC group (ICC versus LCC, HCO_3_^−^: 24.3 (23.1, 25.7) versus 23.5 (22.3, 24.8) mmol/L; BE: -2.39 (-3.73, -1.51) versus − 3.40 (-4.73, -1.91) mmol/L; lactate: 2.2 (1.9, 3.3) versus 2.5 (2.0, 4.3) mmol/L; Hb: 14.6 (13.9, 15.4) versus 14.9 (13.9, 16.3) g/dl; *P* < 0.05). No differences were found in pH, pCO_2_, pO_2_, Hct and glucose between two groups (Table 2).

### Non-diabetic mothers versus diabetic mothers in ICC or LCC

There were no differences in acid-base status and glucose of umbilical arterial blood between the two groups in both ICC and LCC. Among the neonates received ICC, the Hb and Hct levels were obviously higher in DM group than those in NDM group (DM versus NDM, Hb: 15.2 (14.0, 16.1) versus 14.6 (13.9, 15.4) g/dl, *P* = 0.007; Hct: 44.0 (39.8, 47.6) versus 42.5 (39.9, 44.8) %, *P* = 0.02). However, the differences disappeared among neonates who received LCC.

## Discussion

Our study showed significant alterations of partial umbilical arterial blood gas parameters both in neonates with non-diabetic mothers and diabetic mothers after a delay of cord clamping more than 30 s after birth. Later cord clamping of ≥ 30 s was associated with reduced HCO_3_^−^ and BE and increased lactate, without variations in arterial pH, pCO_2_ and pO_2_, suggesting a tendency towards metabolic acidosis. Umbilical arterial blood acid-base state in DM was similar with that in NDM both in ICC group and LCC group. Hb and Hct levels in umbilical arterial blood were higher in DM group than those in NDM group when the neonates received ICC rather than LCC.

Two prospective observational studies used blood samples from unclamped umbilical cords to investigate umbilical cord blood gas at different time points after birth in healthy full-term newborns [7, 9]. Wiberg et al. enrolled 66 neonates delivered vaginally and found that there was a trend of mixed acidosis in umbilical artery (pH, HCO_3_^−^ and BE decreased, and pCO_2_, pO_2_ and lactate increased) from birth to 90 s of life, with the most obvious changes in the first 45 s [7]. Another study showed that 3-minute LCC also resulted in decreased pH, HCO_3_^−^ and BE and increased pCO_2_, pO_2_ and lactate during cesarean section, and similar changes during vaginal delivery except for pCO_2_ [9]. Likewise, we found that HCO_3_^−^ and BE were higher and lactate was lower after LCC of more than 30 s in both NDM and DM group, but there was no change in arterial pH and CO_2_. It might be related to different methods of sampling (samples from an unclamped cord versus samples from a double clamped cord).

Factors including the position of fetus, delivery mode, anesthesia method could influence cord gas parameters [2]. Selective cesarean section led to increased pH, BE, HCO_3_^−^ and pO_2_, and reduced pCO_2_, similar to adult blood gas values [17]. Spinal anesthesia was thought to increase the risk of cord acidemia [18]. In this study, all neonates were in vertex presentation, and all mothers received epidural anesthesia in cesarean section or received epidural labor analgesia during vaginal delivery. The ratio between vaginal delivery and cesarean section was similar in each group. Thus, cord gas parameters were comparable among groups.

Hidden acidosis might account for the umbilical arterial acid-base imbalance both in NDM and DM after later cord clamping. During cesarean section, both blood obstruction in the inferior vena cava caused by maternal supine position and anesthesia resulted in placental hypoperfusion [19, 20]. During vaginal delivery, uterine contraction intermittently interrupted the utero-placental circulation. These led to transient fetal hypoxia. Blood transported into central organs in priority, and peripheral organs with reduced blood supply underwent anaerobic metabolism, a phenomenon known as “hidden acidosis”. LCC carried more blood to neonates, peripheral organs were preferentially perfused, and acid metabolites were released into circulation [21], which might be the reason for the detected increased lactate and decreased HCO_3_^−^ and BE in our study.

The lung perfusion increases from 8 to 45% of the cardiac output immediately after birth [22]. After the establishment of effective lung ventilation, CO_2_, as volatile acid, can decrease rapidly. In contrast, the fixed acids continue to accumulate, resulting in decreasing bicarbonate and BE [7]. In our study, neonates with LCC of ≥ 30 s already had adequate lung ventilation, enabling CO_2_ to transport quickly out of the body. Thus, we observed an increase in lactate, which led to a decrease in HCO_3_^−^ and BE. But CO_2_ remained at the same level as the neonates who received ICC.

Base excess, rather than pH, is an ideal indicator of the degree of hypoxia. BE depends linearly on the accumulation of metabolic acid, while large changes in hydrogen ion concentration are manifested only by small changes in pH. Lactate detected in umbilical cord blood comes almost entirely from the fetus [2]. Thus, lactate and BE are more appropriate indexes to evaluate neonatal acid-base status. Alterations in blood gas combined with abnormal symptoms such as low Apgar score could provide more reliable evidence in predicting adverse perinatal outcomes [2]. Whereas, for ethical reasons, we enrolled neonates with Apgar score of > 7 who did not require immediate resuscitation for implementing LCC. Hence, our findings of decreased BE and increased lactate after LCC of ≥ 30 s seemed to have no significance in predicting the prognosis of neonates.

The effect of LCC on umbilical arterial pO_2_ remained inconclusive. De Paco et al. and Andersson et al. demonstrated that LCC increased umbilical arterial pO_2_ because of a continuing supply of oxygen [10, 11]. However, pO_2_ was not affected in another study [12]. Our study revealed a slight non-significant increase in pO_2_ after LCC of ≥ 30 s compared with ECC (The median clamping time: 6 s) in both NDM and DM. This may be related to the fact that most neonates were able to establish good ventilation immediately after birth. In addition, neonates of DM are at increased risk of transient shortness of breath [13]. The increase in pO_2_ caused by LCC was offset by the decrease in pO_2_ caused by abnormal pulmonary ventilation in neonates of DM [13].

Neonates of insulin-dependent diabetic mothers were at high risk of perinatal asphyxia. Analysis of umbilical blood samples from neonates of type 1 diabetic mothers in later pregnancy showed acidosis and hyperlacticaemia, without hypoxemia [23]. Intrauterine fetal death in diabetic mothers might be due to metabolic acidosis, rather than hypoxia [23], because fetal hyperglycemia and subsequent hyperinsulinemia caused by maternal hyperglycemia could increase metabolic activity and lactic acid accumulation in the body of the fetus. Our data showed that compared with neonates of NDM, neonates of DM had no change in umbilical cord blood gas parameters, whether they received ICC or LCC. This might be because the blood glucose was well-controlled through diet alone in most diabetic mothers and the umbilical artery blood glucose levels at birth were similar in NDM and DM in this study. Another reason was that neonates with suspicious fetal distress and Apgar score of ≤ 7 were excluded.

Gestational diabetes is associated with neonatal polycythemia [24–26], possibly due to elevated insulin and erythropoietin [13]. This study was the first to use umbilical cord blood gas to find an increase in hematocrit and hemoglobin in neonates of DM after ICC. However, after LCC, there were no differences in hematocrit and hemoglobin between neonates of NDM and DM, possibly because LCC delivered more blood to neonates, thereby eliminating these differences.

In this study, hemoglobin, hematocrit and glucose levels were tested in umbilical cord blood. Previous studies found that hemoglobin and hematocrit were higher in neonatal blood than those in umbilical cord blood after birth [27], and plasma glucose was lower [28]. Despite of these differences, three indexes in cord blood were all positively correlated with those in neonatal blood [27, 28]. Thus, the umbilical cord blood could be alternative in exploring neonatal hematologic status.

This study was the first one to report on umbilical arterial blood gas in neonates of diabetic mothers at cord clamping ≥ 30 s. We found that the acid-base status of neonates of DM was similar to that of the healthy neonates of NDM at birth. A delay of cord clamping for ≥ 30 s was associated with a tendency towards metabolic acidosis in neonates of DM. In clinical practice including vaginal delivery and cesarean section, for neonates of diabetic mothers without the need for immediate resuscitation, especially those whose mothers have well-controlled blood glucose during pregnancy, cord clamping ≥ 30 s could be adopted. Whereas, several limitations should be considered in our study. First, we merely explored the effect of LCC on umbilical cord blood gas without exploring the safety of LCC, such as neonatal bilirubin and phototherapy rate. Second, most mothers in our study were with well-controlled blood glucose. Further studies focusing on insulin-dependent mothers are needed. Third, partial cases with blood gas parameters missing led to an inevitable bias. Lastly, this study examined LCC for just 30 s or more and not for longer periods.

In summary, cord clamping ≥ 30 s was associated with a trend of metabolic acidosis in umbilical arterial blood both in neonates of diabetic mothers and non-diabetic mothers, most likely due to “hidden acidosis”. Umbilical arterial blood gas parameters at birth were similar in DM and NDM.

## Data Availability

The datasets used and/or analysed during the current study available from the corresponding author on reasonable request.

## Abbreviations

BE: Base excess
DM: Diabetic mothers
HCO_3_^-^: Bicarbonate
ICC: Immediate cord clamping
LCC: Later cord clamping
NDM: Non-diabetic mothers
pCO_2_: Partial pressure of carbon dioxide
pO_2_: Partial pressure of oxygen
